# Prevalence of anaemia among patients with heart failure at the Brazzaville University Hospital

**DOI:** 10.5830/CVJA-2015-021

**Published:** 2015

**Authors:** Méo Stéphane Ikama, Bernice Mesmer Nsitou, Ngamami Solange Mongo, Gisèle Kimbally-Kaky, Jean Louis Nkoua, Innocent Kocko

**Affiliations:** Cardiologie, CHU de Brazzaville, Brazzaville, Congo; Cardiologie, CHU de Brazzaville, Brazzaville, Congo; Cardiologie, CHU de Brazzaville, Brazzaville, Congo; Cardiologie, CHU de Brazzaville, Brazzaville, Congo; Cardiologie, CHU de Brazzaville, Brazzaville, Congo; Hématologie clinique, CHU de Brazzaville, Brazzaville, Congo

**Keywords:** heart failure, anaemia, prevalence, prognosis, Congo

## Abstract

**Background:**

Heart failure (HF) is a frequent cause of hospitalisation in cardiology. Its prognosis depends on several risk factors, one of which is anaemia.

**Objectives:**

We aimed to determine the prevalence of anaemia in patients with heart failure, and evaluate its impact on their prognosis.

**Methods:**

This article describes a cross-sectional study with prospective collection of data, carried out from 1 January to 31 December 2010 in the Department of Cardiology at Brazzaville University Hospital, Congo. Patients admitted for heart failure were included. Anaemia was defined as a haemoglobin level < 12 g/dl for men and < 11 g/dl for women.

**Results:**

In total, 130 men (47.8%) and 142 women (52.2%) were recruited, mean age 56.9 ± 16.5 years. The prevalence of anaemia was 42%. Average levels of haemoglobin were 9.4 ± 1.8 and 13.8 ± 4.9 g/dl for the anaemic (A) and non-anaemic (NA) patients, respectively (*p* = 0.0001). Two hundred and forty-nine patients (91.5%) were in NYHA functional class III–IV. Forty-seven patients (17.3%) were on oral anticoagulation and 15 (5.5%) were on aspirin. The average duration of hospital stay was 19.1 ± 16.7 days, without a significant difference between the A and NA groups (19.4 ± 12 vs 18.8 ± 13.8 days; *p* = 0.79, respectively). Total mortality rate was 17%, with a significant difference between the A and NA groups (26 vs 10%; *p* = 0.001).

**Conclusion:**

This preliminary study showed a high prevalence of anaemia in patients with heart failure, and it had a negative effect on the prognosis.

## Abstract

Heart failure (HF) is a frequent cause of hospitalisation in cardiology. Its prognosis depends on several factors, including anaemia, which is common among patients with heart failure.^1^ Anaemia is an independent prognostic factor for mortality in chronic HF and is associated with higher rates of mortality, hospitalisation and re-admission.^2^,^3^ Anaemia is a powerful independent predictor of death and hospitalisation in systolic and diastolic dysfunction.^2^,^4^-^7^

In order to improve the management of patients suffering from systolic and diastolic HF, it is critical to understand the relationship between HF and anaemia, and the possible outcomes. The aim of this study was to determine the prevalence of anaemia in patients with heart failure and to evaluate its impact on the prognosis of patients in Brazzaville, Congo.

## Methods

This article describes a cross-sectional study with a prospective approach to data collection, carried out from 1 January to 31 December 2010 in the Department of Cardiology and Internal Medicine at Brazzaville University Hospital. The study included patients admitted for left or biventricular heart failure. Patients admitted for exclusively right heart failure, or a cause other than heart failure, as well as for sickle anaemia, were excluded.

Anaemia was defined as a haemoglobin level < 12 g/dl for men and < 11 g/dl for women. Two hundred and seventy-two patients were selected and divided into two groups according to anaemic status: anaemic (*n* = 114) and non-anaemic patients (*n* = 158).

Socio-demographics such as age, gender and socio-economic level were analysed, as well as clinical and echocardiographic parameters, including type of heart failure (left or biventricular), NYHA (New York Heart Association) functional class, the use of aspirin and/or oral anticoagulation, type of heart disease, and left ventricular ejection fraction (LVEF). In addition, we studied blood profiles, including haemoglobin level, renal function (estimated by glomerular filtration rate using the Cockroft–Gault equation; considered to be lowered if GFR < 60 ml/min). Finally, we analysed prognosis in terms of duration of hospital stay, and mortality rate (outcome for that same admission).

## Statistical analysis

The data were analysed with Epi-info 3.5.1 software. The chi-squared and ANOVA tests allowed the comparison of qualitative and quantitative variables, respectively. The significance level was *p* < 0.05.

## Results

A total of 272 patients were evaluated, including 130 men (47.8%) and 142 women (52.2%), with a mean age of 56.9 ± 16.5 years (range: 18–97). The prevalence of anaemia was 42%, with an average haemoglobin level of 11.9 ± 4.4 g/dl (range: 4.7–15.2). The average haemoglobin levels were 9.4 ± 1.8 and 13.8 ± 4.9 g/l in the anaemic (A) and non-anaemic (NA) patients, respectively (*p* = 0.0001). The main patient characteristics are shown in Table 1.

**Table 1:** Patient characteristics

*Parameters*	*Patients (n = 272)*
Male gender, n (%)	130 (47.8)
Age (years), SD (range)	56.9 ± 16.5 (18–97)
Low socio-economic level, n (%)	211 (77.5)
HIV +, n (%)	12 (4.4)
Biventricular HF, n (%)	233 (85.7)
NYHA III–IV, n (%)	249 (91.5)
Haemoglobin (g/dl), SD (range)	11.9 ± 4.4 (4.7–15.2)
Aspirin, n (%)	15 (5.5)
Oral anticoagulation, n (%)	47 (17.3)
LVEF (%), SD (range)	49.3 ± 14.7 (22–75)
Hospitalisation stay (days), SD	19.1 ± 16.7
Mortality rate, n (%)	46 (17)

HIV: human immunodeficiency virus; HF: heart failure; NYHA: New York Heart Association; LVEF: left ventricular ejection fraction.

Heart failure was biventricular in 233 cases (85.7%) and left HF in 39 cases (14.3%). Two hundred and forty-nine patients (91.5%) were in NYHA functional class III–IV, with no difference between the A and NA patients (*p* = 0.6). The heart diseases diagnosed were hypertensive heart disease in 106 cases (39.0%), dilated cardiomyopathy in 86 cases (31.6%), myocarditis in 27 cases (9.9%), valvular heart disease in 24 cases (8.8%), ischaemic heart disease in 15 cases (5.5%), and unspecified cause in 14 cases (5.1%).

Average left ventricular ejection fraction was 48 ± 14.6% in A and 51.3 ± 15% in NA patients (*p* = 0.43). Average glomerular filtration rate was 54.6 ± 12.5 ml/min in A and 70.4 ± 10.2 ml/min in NA patients (*p* = 0.004). Forty-seven patients (17.3%) were on oral anticoagulation and 15 (5.5%) were on aspirin.

The average duration of hospital stay was 19.1 ± 16.7 days, with no statistical difference between the A and NA patients (19.4 ± 12 vs 18.8 ± 13.8 days, respectively; *p* = 0.79). Total mortality rate was 17%, with a significant difference between the A and NA patients (26 vs 10%; *p* = 0.001). The comparison between A and NA patients is given in Table 2.

**Table 2:** Comparison between anaemic and non-anaemic patients

*Parameters*	*Anaemic patients (n = 114)*	*Nonanaemic patients (n = 158)*	*p-value*
Age (years)	54.9 ± 18.3	58.3 ± 15.1	0.105
Haemoglobin (g/dl)	9.4 ± 1.8	13.8 ± 4.9	0.0001
Biventricular HF, n (%)	101 (43.3)	0.0001	0.159
NYHA III–IV, n (%)	106 (93)	143 (90.5)	0.6
Aspirin, n (%)	3 (2.5)	12 (7.6)	0.06
Oral anticoagulation, n (%)	19 (16.7)	28 (17.7)	0.47
LVEF (%)	48 ± 14.6	51.3 ± 14.9	0.43
Glomerular filtration rate (ml/min)	54.6 ± 12.5	70.4 ± 10.2	0.004
Hospitalisation stay (days)	19.4 ± 12	18.8 ± 13.8	0.79
Mortality rate, n (%)	30 (26)	16 (10)	0.001

HF: heart failure; NYHA: New York Heart Association; LVEF: left ventricular ejection fraction.

## Discussion

It has been shown that advanced age is a predictive factor of a strong prevalence of anaemia in heart failure.^6^,^8^ In our study, the patients were relatively young, with an average of 57 years, in comparison with large series in developed countries, where the median age of patients was 70 years.^9^,^10^ In Africa, very few studies have been conducted assessing anaemia in HF patients.^11^-^13^

In our study, the prevalence of anaemia in HF was 42%, near to the 49% that was found in France by Abassade *et al.*,^10^ and lower than the 64.3% found by Kuule *et al.* in Uganda.^11^ In the literature, the prevalence of anaemia is variable, from 4 to 61%, with the majority of studies finding it between 18 and 20%.^14^-^16^ This large variability may be explained by methodological differences, due mainly to the definition of anaemia.^2^,^3^,^17^-^19^

Most publications use the definition of anaemia by the World Health Organisation (anaemia is a haemoglobin concentration < 13 g/dl in men and < 12 g/dl in postmenopausal women), and by National Kidney Foundation (anaemia is a haemoglobin concentration < 12 g/dl in both men and postmenopausal women).^20^,^21^ The prevalence of anaemia in our study was therefore underestimated; it would have been higher if the WHO criteria for definition had been used.

In chronic HF, factors associated with a high prevalence of anaemia include concomitant kidney disease, advanced age, female gender, African American ethnicity, diabetes, hypertension, and lower estimated glomerular filtration rates.^8^,^5^,^22^ In our study, the aetiological research on anaemia was not systematic.

In general, the aetiology of anaemia in chronic HF is multifactorial, and multiple mechanisms contribute to anaemia in chronic HF:^15^,^23^ iron and other haematological deficiencies, renal insufficiency, the role of haemodilution, chronic diseases and ‘inflammation’, and the renin–angiotensin system. Iron deficiency appears to be the most common cause of anaemia in HF.^24^,^25^ In the African context,^26^ malnutrition, infectious pathology (intestinal parasites, HIV infection), and the congestive nature of HF (salt and water retention, advanced chronic HF) may partially explain the prevalence of anaemia in African subjects, the majority being hypertensive and potentially renal insufficient.

A large number of studies have confirmed that anaemia is a strong, independent predictor of increased mortality rate and hospitalisation stay in patients with systolic and diastolic dysfunction, new-onset HF, and severe chronic HF.^2^,^4^-^7^,^24^ In our study, these reports were confirmed in terms of higher mortality rate, and longer hospital stay in the anaemic patients compared to non-anaemic sunjects.

## Conclusion

This preliminary study showed a high prevalence of anaemia in chronic HF patients and its negative impact on the prognosis (high mortality rate, longer hospitalisation) of patients. The prognosis of anaemic patients suffering from HF may be improved by treatment of the anaemia.
